# Amplification of annual and diurnal cycles of alpine lightning

**DOI:** 10.1007/s00382-023-06786-8

**Published:** 2023-04-27

**Authors:** Thorsten Simon, Georg J. Mayr, Deborah Morgenstern, Nikolaus Umlauf, Achim Zeileis

**Affiliations:** 1 Department of Mathematics, Universität Innsbruck, Technikerstrasse 21a, 6020 Innsbruck, Austria; 2 Department of Statistics, Universität Innsbruck, Universitätsstrasse 15, 6020 Innsbruck, Austria; 3 Department of Atmospheric and Cryospheric Sciences, Universität Innsbruck, Innrain 52, 6020 Innsbruck, Austria

**Keywords:** Lightning location system, ERA5, Climate change, Generalized additive models, Machine learning, Complex terrain

## Abstract

The response of lightning to a changing climate is not fully understood. Historic trends of proxies known for fostering convective environments suggest an increase of lightning over large parts of Europe. Since lightning results from the interaction of processes on many scales, as many of these processes as possible must be considered for a comprehensive answer. Recent achievements of decade-long seamless lightning measurements and hourly reanalyses of atmospheric conditions including cloud micro-physics combined with flexible regression techniques have made a reliable reconstruction of cloud-to-ground lightning down to its seasonally varying diurnal cycle feasible. The European Eastern Alps and their surroundings are chosen as reconstruction region since this domain includes a large variety of land-cover, topographical and atmospheric circulation conditions. The most intense changes over the four decades from 1980 to 2019 occurred over the high Alps where lightning activity doubled in the 2010 s compared to the 1980 s. There, the lightning season reaches a higher maximum and starts one month earlier. Diurnally, the peak is up to 50% stronger with more lightning strikes in the afternoon and evening hours. Signals along the southern and northern alpine rim are similar but weaker whereas the flatlands surrounding the Alps have no significant trend.

## Introduction

Lightning has recently been added as an essential climate variable of the Global Climate Observing System (Belward et al. 2016; Aich et al. 2018). Cloud-to-ground lightning strikes may damage equipment and structures such as wind turbines (Becerra et al. 2018) and power lines (Cummins et al. 1998), start fires (Reineking et al. 2010) and injure or kill people (Ritenour et al. 2008; Holle 2016). Further, lightning contributes NO$_x$ and ozone as air pollutants (DeCaria et al. 2005; Zhang et al. 2020), and lightning threatens permafrost (Finney 2021).

Since lightning is an essential climate variable, there is an urgent need to understand how lightning has been evolving over the past decades. This is challenging as lightning is affected at various temporal and spatial scales from micro physics (Houze 2014) over meso-scale dynamics (Feldmann et al. 2021) to synoptics (Piper and Kunz 2017; Posch 2018). These scales are all subject to different changes by a changing climate.

We aim to investigate the historic evolution of cloud-to-ground lightning across the European Eastern Alps. Climatologies show that this region is a hot spot within Europe (Poelman et al. 2016; Enno et al. 2020; Taszarek et al. 2020). The interactions of the complex terrains and atmospheric processes such as circulation and radiation lead to persistent forcings (Bertram and Mayr 2004). Orographic lifting, thermally induced circulations (plains–mountains, slope winds, valley winds), and lee effects can all trigger lightning in this region (Houze 2012). Further, this region is exposed to climate change in a special way as the area covered by snow and ice is decreasing (Matiu et al. 2021) warming is more pronounced than in other parts of Europe (Brugnara 2020), and convective precipitation increases at high elevations (Giorgi et al. 2016). A trend analysis of the mean annual number of days with thunderstorms since 1979 indicate an increase over the Alps (Taszarek et al. 2019).

Leveraging recent advancements in three fields enables us to tackle the reconstruction of lightning over a 40 year time period: (1) Decade-long homogeneously detected *lightning observations* (Cummins and Murphy , 2009; Schulz et al. , 2016). (2) Physically-based numeric *model outputs* for hourly reanalysis of the atmospheric conditions including micro-physics over several decades (Hersbach et al. 2020). (3) *Flexible regression techniques* from statistics and machine learning (see Hastie et al. 2009, for an overview) that allow to select and link the relevant model outputs to the lightning observations in order to obtain reconstructions. Note that all three elements have to come together to yield a high-quality answer. Just using detected lightning observations over a decade would be too short to assess climate. The physical models alone do not resolve thunderstorms. And the statistical/machine learning techniques are needed for obtaining a satisfactory regression fit with good out-of-sample predictive performance.

Previous studies applied proxies for lightning to assess its historic behavior from atmospheric reanalyses. Classically, cloud top height (Price and Rind 1992), the product of CAPE and precipitation (Romps et al. 2018), the square root of CAPE multiplied by deep layer shear (Taszarek et al. 2019), CAPE exceeding a threshold conditioned on the occurrence of convective precipitation (Taszarek et al. 2021), or vertical iceflux (Finney et al. 2014) have been used. Moreover, the content of such proxies depends on the micro-physical parameterizations used in the numerical models (Charn and Parishani 2021). In sum the current scientific status it that different lightning proxies and/or micro-physical parameterizations can lead to contrary results in the light of a changing climate. For instance, using only iceflux could project a decrease of future lightning (Finney et al. 2018), whereas the product of CAPE and precipitation projected an increase of lightning under climate change (Romps et al. 2014). Further, discussion on this controversy can be found in Murray (2018) and Taszarek et al. (2021).

The aim of this study is to come up with a description of lightning composed of diverse processes and use this description to analyze historic lightning. In a first step, a flexible regression model links the detected lightning observations to the model output from the atmospheric reanalyses when both data sources overlap (2010–2019). The interest lies in whether *lightning occurred*, not (yet) in the number of flashes per time and area (which is e.g. modeled in Simon et al. 2019). The *occurrence* of cloud-to-ground lightning could also be interpreted as the occurrence of thunderstorms or the likelihood of convective initiation. In a second step, probabilistic reconstructions of lightning occurrence are obtained from the flexible regression model for the overall period 1980–2019 including the period when solely atmospheric model outputs but no lightning detection observations are available.

Similarly, statistical post-processing of output from numerical weather prediction (NWP) models using historical observation records is widely used in the weather forecasting literature and termed *model output statistics* (MOS, Glahn and Lowry 1972). Therefore, we call our approach *lightning-MOS*. While lightning-MOS serves here to reconstruct historic lightning, it could also be used for future projections.

Moreover, other statistical/machine learning approaches have also been used in previous lightning studies. In particular, Ukkonen and Mäkelä (2019) have evaluated various machine learning classifiers for linking reanalysis output to the occurrence of lightning. Although they have not computed reconstructions with their classifiers, they encouraged using this approach to study climate trends. Beyond lightning, GAMs have proven their capabilities for severe weather trends (Rädler et al. 2018). Similar machine learning approaches have been used to generate wildfire danger maps (Vitolo et al. 2020; Coughlan et al. 2021). Also, the field of post-processing numerical weather predictions leverages such techniques ranging from GAMs combined with objective variable selection (Simon et al. 2018) to deep neural networks (Kamangir et al. 2020).

The outline of the remainder of the paper is structured as follows: Using ALDIS lightning detection data (Sect. 2.1) and the ERA5 atmospheric reanalyses (Sect. 2.2) the lightning-MOS (Sect. 3.1) reconstructs 40 years (1980–2019) of consistent lightning data for the European Eastern Alps. In particular, it yields the probability for the occurrence of lightning in an ERA5 grid cell within an hour. With these data on hand an analysis (Sect. 4) of the spatio-temporal variability over the last 40 years is conducted. The analysis focuses on identifying potential shifts and/or expansions of diurnal cycles and the thunderstorm seasons (Sect. 4.3), as well as the investigation of climate trends over the past 40 years (Sect. 4.4). Section 5 discusses the approach and Sect. 6 provides the conclusions.

## Data

**Fig. 1:**
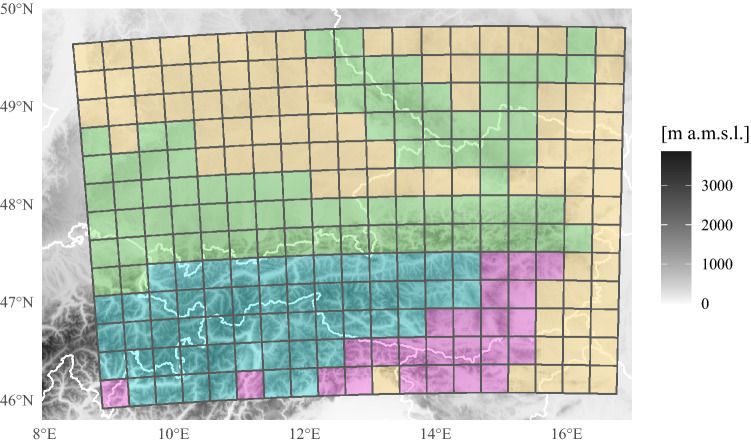
Topography of study area. The data are aggregated to a $32~\textrm{km}\,\times \,32~\textrm{km}$ mesh. Colored grids indicate the subdomains. turquoise: High Alps (HIA), green: Northern Alpine rim and northern mountain ranges (NAR), purple: Southern Alpine rim (SAR), yellow: Surrounding flatlands (FLT)

Two data sets were utilized for the purpose this study: Measurements from the lightning location system ALDIS (Sect. 2.1), and direct and derived parameters from ERA5 (Sect. 2.2).

### Lightning detection data

The Austrian Lightning Detection & Information System (ALDIS) is part of the European effort EUCLID (Schulz et al. 2016). Lightning is detected using also information from EUCLID sensors outside of the ALDIS domain in order to ensure a spatially homogeneous detection efficiency. To ensure temporal homogeneity, the period 2010–2019 is chosen, for which changes to software and hardware have had a negligible effect on detecting cloud-to-ground flashes (Poelman et al. 2016). The assumption of temporal homogeneity is in particular valid as from these measurements the binary information is extracted whether at least one cloud-to-ground flash exceeding some current limits occurred within spatio-temporal grid cells of $32\,\textrm{km}\,\times 32\,\textrm{km}$ and one hour. A current threshold filter from $-2$ to $+15$ kA serves to eliminate intracloud pulses that are often misclassified as cloud-to-ground flashes (Poelman et al. 2016).

Finally, detected and filtered cloud-to-ground flashes are hourly aggregated to a grid with a 32 km spatial mesh. Each grid cell with at least one cloud-to-ground flash is labelled as *lightning cell*. Spatially, the grid ranges from $8.5^\circ$ to $16.9^\circ$ E and from $45.9^\circ$ to $49.8^\circ$ N (Fig. 1). Temporally, the lightning data was homogeneously detected and processed from 2010 to 2019. From this period of time data covering the boreal summers (Apr–Sep) are used, which is the main lightning season in European Alps.

### Atmospheric reanalysis output

ECMWF’s fifth reanalysis (ERA5, Hersbach et al. 2020) comes with a resolution of 32 km horizontally and of 1 h in time. Further, its vertical discretization with 137 levels resolves many details valuable for the description of convective processes. We pre-select 40 single level variables and augment these by deriving another 45 variables from vertical profiles within the troposphere, for which the 74 model levels between the surface (level 137) and approximately 15 km altitude (or 120 hPa at level 64) are used.^1^

The focus of the pre-selection of single level variables and the derivation of further variables from model levels is on covering processes relevant for convection and lightning from atmospheric environments favorable for the formation of thunderstorms to electrical charge separation. Examples are cloud top height, wind shear within and below the clouds, the amount of liquid and solid water between predefined isotherms, and vertical iceflux in the mid-troposphere. The idea of this pool of potential variables is that it includes a fair amount of variables that are likely to be important for the description of lightning. Uninformative variables will be filtered out by the objective selection algorithm (Sect. 3.1).

The data between 2010 and 2019 are used for training and evaluation of the lightning-MOS, data from 1980 onward are used for the probabilistic reconstruction of lightning occurrences.

### Subdomains

The $32\,\textrm{km}\,\times 32\,\textrm{km}$ cells are grouped into four subdomains with similar characteristics of topographically induced lifting. Figure 1 shows the assignment of the cells and Table 1 summarizes their properties. Flatlands with little altitudinal variation and ERA5 altitudes below $500\,\mathrm{m~a.m.s.l.}$ are regions without any significant lifting. The northern rim of the Alps and Bavarian and Bohemian forest further north and similarly the southern rim of the Alps are regions where impinging air first encounters steeper slopes and the ensuing topographical lifting might release convective instabilities. The even higher topography with ERA5 altitudes above $1200\,\mathrm{m~a.m.s.l.}$ beyond the first topographic lifting by the enclosing rims is the fourth subdomain.

**Table 1:** Properties of subdomains shown in Fig. 1

	Description	Model altitude	Spatial coverage ($\textrm{km}^2$)
HIA	High Alps	$>\,1\,200\,\textrm{m}$	54,272
NAR	Northern Alpine rim and mountain ranges	$>\,500\,\textrm{m}$ & $<\,1200\,\textrm{m}$	81,920
SAR	Southern Alpine rim	$>\,500\,\textrm{m}$ & $<\,1200\,\textrm{m}$	24,576
FLT	Surrounding flatlands	$<\,500\,\textrm{m}$	92,160

## Methods

This section introduces the flexible regression approach that links the detected ALDIS lightning observations to the ERA5 output. We will denote this approach as *lightning MOS* which builds on generalized additive models and objective variable selection (Sect. 3.1). Further, this section presents reference approaches. The first is a climatology that describes lightning occurrence conditioned on the spatio-temporal setting. The remaining references are three common proxies of lightning and convective environments (Sect. 3.2).

### Lightning model output statistics

The lightning-MOS explains the observed lightning occurrence with the parameters from ERA5 using a flexible regression framework. A generalized additive model (GAM, Wood 2017) identifies non-linear effects between the two sets of data. The lightning-MOS is set up using a GAM to model $\pi$ which is the probability of lightning occurrence such that,

1 $$\begin{aligned} \begin{aligned} \text {logit}(\pi ) =&\beta _0 + f_1(\texttt{lon,lat, hour}) + f_2(\texttt{yday}) \\&+ f_3(\texttt{topo}) \\&+ g_1\left( \texttt{x}_1\right) + \cdots + g_p\left( \texttt{x}_p\right) . \end{aligned} \end{aligned}$$

The $\text {logit}()$ function on the left-hand side of Eq. 1 maps the probability of lightning $\pi$ to the real line. The right-hand side is composed of the intercept $\beta _0$ which estimates a global average of the regression model and several potentially non-linear functions $f_{*}()$ and $g_{*}()$ that identify deviations from the global average conditioned on the explanatory variables. The non-linear functions $f_*()$ and $g_*()$ are set up using P-splines and thin plate regression splines (Wood 2017).

The three functions $f_{*}()$ describe the effect of the spatio-temporal setting. The first $f_1(\texttt{lon,lat, hour})$ is a three-dimensional smooth effect that allows variations of the diurnal cycle over geographical space. The second $f_2(\texttt{yday})$ and the third $f_3(\texttt{topo})$ add an annual cycle and the effect of topography, respectively.

The functions $g_*()$ model the effects of the ERA5 parameters (Sect. 2.2) for which $\texttt{x}_*$ stands as a placeholder in eq. 1. Which ERA5 parameters and how many are used within the lightning-MOS is not pre-defined but determined objectively by a selection algorithm that searches for the most important ERA5 parameters to explain the probability of lightning occurrence $\pi$. The algorithm for the objective selection of ERA5 parameters is a combination of gradient boosting (Bühlmann and Hothorn 2007) and stability selection (Meinshausen and Bühlmann 2010) and will be introduced briefly. A detailed description of the algorithm can be found in Simon et al. (2018) who developed the algorithm for a selection of NWP parameters for a thunderstorm forecasting scheme.

The selection of the most important non-linear functions $g_*()$ is performed using gradient boosting combined with stability selection. Gradient boosting is an iterative gradient descent algorithm, where the function which minimizes the residual sum of squares when fitted to the gradient of the log-likelihood (Eq. 3 in Simon et al. 2018) is slightly updated in each iteration. The estimates converge to the maximum likelihood estimates, when the number of iterations approaches infinity. Early stopping of the iterations ends in regularized estimates of the functions $g_*()$, and also serves as selection procedure when individual functions are equal to 0 at the final iteration.

If gradient boosting is applied as a stand-alone method the number of iterations—and thus the degree of regularization—can be determined by means of information criteria or cross-validation. Here the main purpose of gradient boosting is to select important functions $g_*()$. It is desirable to avoid the selection of numerous non-informative terms. Stability selection is a convenient resampling method for controlling the number of selected non-informative terms by gradient boosting (Meinshausen and Bühlmann, 2010; Hofner et al., 2015).

Rather than applying the boosting algorithm to all observations, stability selection is based on drawing a subsample of the training data, running the boosting algorithm until a predefined number of iterations is reached. This procedure is repeated many times, here 100 times. Afterwards the relative selection frequencies per nonlinear term are computed. Finally, the terms which were selected most frequently are included in the final model (cf. algorithm in Hofner et al. 2015). This final GAM is optimized using restricted maximum likelihood method in order to identify the non-linear functions $f_*()$ and $g_*()$ (Wood et al. 2017).

### Reference models

To assess the performance of the lightning-MOS and to compare it against other approaches describing lightning or thunderstorms two conceptually different types of reference models are taken into account. First, a climatology that describes the occurrence of lightning given the spatio-temporal setting (Simon and Mayr 2022). Second, proxies that are well known for their ability to describe convective environments (Finney et al. 2014; Taszarek et al. 2017, 2021).

A climatology is estimated as a baseline to assess the performance of the lightning-MOS. A GAM (Eq. 1) is set up exclusively with the non-linear functions for spatio-temporal setting $f_*()$ using covariates for geographic space (longitude $\texttt{lon}$ and latitude $\texttt{lat}$), time (day of the year $\texttt{yday}$ and hour of day $\texttt{hour}$) and ERA5 model topography $\texttt{topo}$. As none of the functions with ERA5 parameters $g_*()$ are included in the climatology, this comparison elucidates the improvement brought by lightning-MOS over a climatological forecast.

A comparison with the following three state-of-the-art lightning proxies will demonstrate whether and by how much lightning-MOS outperforms them, too. The three proxies are

- CAPE given convective precipitation, which mimics a situation in which CAPE is released (Taszarek et al. 2021),
- iceflux at 450 hPa, which mimics charge separation (Finney et al. 2014), and
- the product of the square root of 2 times CAPE and deep layer shear (0–5 km layer), called capeshear which is known to be a good indicator to distinguish between non-severe and severe thunderstorms (Taszarek et al. 2017).

These quantities are converted into binary yes/no variables by setting thresholds. If the threshold is exceeded the variable indicates the occurrence of lightning. The thresholds are computed such that for each binary proxy variable the fraction of lightning cases throughout the domain and season equals the fraction of lighting cases in the observations. The observations are again the ALDIS cloud-to-ground flashes transformed to binary information on the scale of the ERA5 grid. Following this procedure, the threshold For CAPE-released is $260\,\textrm{J}\,\textrm{kg}^{-1}$, for iceflux the threshold is $1.65\,\times \,10^{-5}\,\textrm{kg}_{\textrm{ice}}\textrm{m}^{-2}_{\textrm{cloud}}\textrm{s}^{-1}$, and for capeshear the threshold is $740\,\textrm{J}^{0.5}\,\textrm{m}\,\textrm{kg}^{-0.5}\,\textrm{s}^{-1}$. Since these thresholds are adapted to our domain, they will likely differ from the values used in other studies.

## Results

Four different topics of results are presented in detail: First, the objectively selected ERA5 parameters for the lightning-MOS (Sect. 4.1). Second, a comparison against observations and the reference approaches (Sect. 4.2). Third, the evolution of diurnal and annual cycles over the past decades (Sect. 4.3). Fourth, the climate trends and their spatial distribution (Sect. 4.4).

### Selected variables

An objective selection algorithm (gradient boosting with stability selection, see Sect. 3.1) is applied to select the most important variables from the pool of ERA5 parameters (Sect. 2.2). Though gradient boosting alone could serve as selection tool, stability selection is used to reduce the chance of selecting non-informative parameters even further. Therefore, the gradient boosting is repeated 100 times on random subsamples each containing 20% of the training data. Finally, only the ERA5 parameters that were selected in at least 99% of the individual boosting runs enter the final model. The algorithm selected 9 parameters (Table 2) from the 85 ERA5 parameters, which are either directly available in ERA5 or derived from ERA5 model level data.

**Table 2:** ERA5 parameters included in the lightning-MOS, which are automatically selected from 85 ERA5 parameters (Sect. 2.2) by the statistical learning algorithm (Sect. 3.1)

Type	Abbreviation	Description
ERA5 single level	$\texttt{cape}$	Convective available potential energy
variables	$\texttt{cp}$	Convective precipitation
	$\texttt{ishf}$	Instantaneous surface sensible heat flux
	$\texttt{mcc}$	Medium cloud cover
	$\texttt{2t}$	2 meter temperature
	$\texttt{tcslw}$	Total column supercooled liquid water
Derived variables	$\texttt{cswc2040}$	Mass of specific snow water content between the $-20^{\circ }C$ and $-40^{\circ }C$ isotherms
	$\texttt{cth}$	Cloud top height in height above ground
	$\texttt{wvc1020}$	Mass of water vapor between the $-10^{\circ }C$ and $-20^{\circ }C$ isotherms

The electrification of clouds requires a mixed phase cloud with differently sized particles as micro-physical condition and strong enough motions to form, collide and separate them as a dynamical condition (e.g. Rakov and Uman 2003). The algorithm selected the snow water content between the $-20^{\circ }C$ and $-40^{\circ }C$ isotherms ($\texttt{cswc2020}$), total column supercooled liquid water ($\texttt{tcslw}$), mass of water vapor between the $-10^{\circ }C$ and $-20^{\circ }C$ isotherms and mid-level cloud cover ($\texttt{mcc}$) and convective precipitation ($\texttt{cp}$) to represent the micro-physical condition. Instantaneous surface sensible heat flux ($\texttt{ishf}$), CAPE ($\texttt{cape}$), two meter temperature ($\texttt{2t}$), and cloud top height above ground ($\texttt{cth}$) represent the dynamical condition.

### Performance and validation

A comparison of metrics assesses the performance of the lightning-MOS relative to the baseline climatology. First, the *deviance explained*, a measure for the goodness-of-fit of GAMs that generalises the residual sum of squares, is presented (Sect. 3.1.4 in Wood 2017). Larger values refer to better capability of the model in explaining the variation of the binary target variable. While the baseline climatology fitted to the whole data of ten years yields a deviance explained of $12.4\,\%$, the lightning-MOS results in a deviance explained of $33.5\,\%$. Further, we computed the Receiver Operating Characteristics (ROC) in a ten-fold cross-validation experiment. The area under the ROC curve (AUC) measures the ability of the two methods to discriminate between the target classes *lightning* and *no lightning*. The AUC is evaluated for each hour of day, month and region separately. The median AUC (2.5% quantile, 97.5% quantile) over these values is 0.90 (0.81, 0.97) for the lightning-MOS, which outperforms the baseline climatology with 0.58 (0.41, 0.77).

**Fig. 2:**
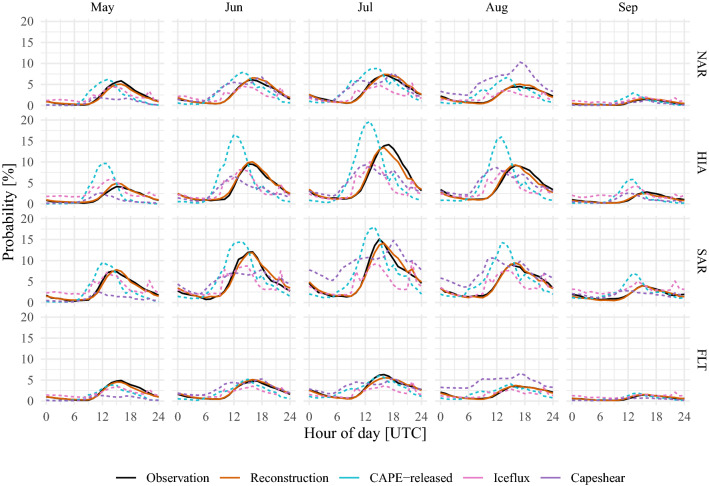
Validation of reconstructed diurnal cycles over the period between 2010 and 2019. The reconstructions are obtained by cross-validation. The dashed lines represent diurnal cycles computed from proxies as discussed in Sect. 5. Columns present diurnal cycles for each month, rows for each region

**Fig. 3:**
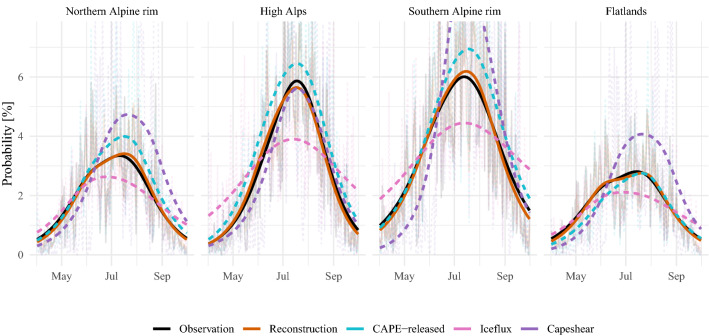
Validation of reconstructed annual cycles over the period between 2010 and 2019. The reconstructions are obtained by cross-validation. The dashed lines represent annual cycles computed from proxies as discussed in Sect. 5. The thick lines in the foreground are a smoothed version of the values for the day of the season (thin lines in the background.)

To further affirm the lightning-MOS, the reconstructed probabilities (ten-fold cross-validation) are compared with the dichotomous observations for the period of 2010–2019. For the comparison of the diurnal cycles (Fig. 2), both the fitted and observed values are aggregated to diurnal cycles for each month and subdomain (NAR, HIA, SAR and FLT). For the comparison of the annual cycles (Fig. 3) the focus is set in the afternoon hours (13–19 UTC) and fitted and observed values are aggregated to each day of the season and subdomain. Since lightning has a strong day-to-day variability, aggregation to the day of the season yields a noisy picture (thin lines in Fig. 3), the values are further smoothed by a cubic spline. The reconstruction (red solid lines in Figs. 2 and 3) closely matches amplitude and phase of the observed diurnal and annual lightning cycles (black solid lines).

Cycles reconstructed with the proxies of CAPE-released, iceflux and capeshear (light blue, pink and purple dashed lines in Fig. 2), on the other hand, deviate strongly from observations. The diurnal cycles obtained from CAPE-released peak about 2–3 h too early. Moreover, the maximum probabilities of this proxy over the high Alps are far too high. The diurnal cycles of the proxy derived from iceflux are not as pronounced as the diurnal cycles of the observations. The probabilities during night and morning are too high and the probabilities during afternoon and evening are too low. Capeshear, similar to CAPE-released, peaks too early. Further, capeshear reveals strong biases in July in the Southern Alpine rim, and in August in SAR, NAR and FLT.

For the comparison of the annual cycles (Fig. 3) fitted and reconstructed probabilities as well as the proxies are averaged for each day of the year and subdomain. CAPE-released captures the annual cycle of the observations very well. Capeshear matches the observed annual cycle in the high Alps nearly completely, but strongly overestimates in the other subdomains, in particular at the end of the season. The iceflux proxy leads to annual cycles with too low an amplitude in the high Alps and the Southern Alpine rim. In the middle of the season it underestimates the observed cycle and at the bounds of the season it overestimates the observed cycle.

### Reconstructed diurnal and annual cycles

**Fig. 4:**
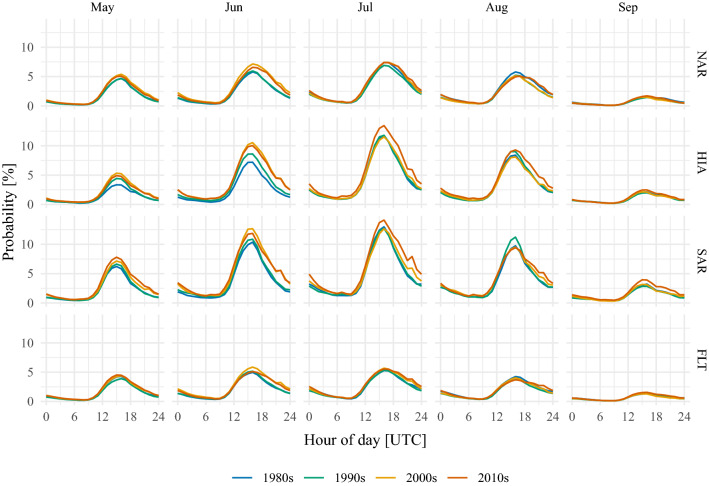
Reconstructed diurnal cycles of probabilities for lightning events averaged over the four decades from 1980s to 2010s (color coded). Columns present diurnal cycles for each month, rows for each region

**Fig. 5:**
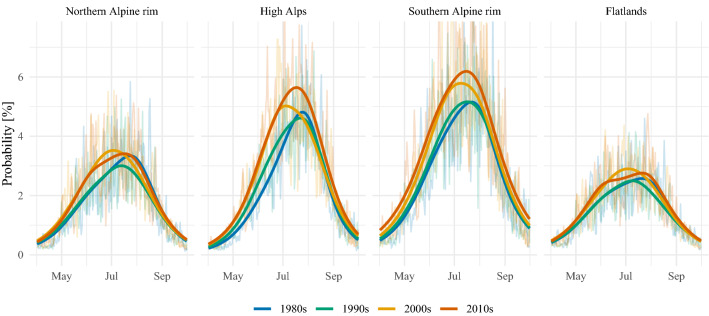
Reconstructed annual cycles of probabilities for lightning events averaged over the four decades from 1980s to 2010s (color coded). The light curves in the background are aggregations to the day of the year. The dark curves in the foreground are smoothed versions of the light curves

The lightning-MOS is used to reconstruct lightning prior to the beginning of homogeneous lightning data. To investigate the diurnal cycles, the reconstructed probabilities are averaged for each hour, month and subdomain. Figure 4 displays the results by decade. The 1980s are in blue, the 1990s in green, the 2000s in yellow, and the 2010s in red. The curves for the annual cycle in (Fig. 5) are obtained from aggregating the probabilities for each day of the year and subdomain and further temporal smoothing to account for the strong day-to-day variability of lightning (Simon et al. 2017).

Zooming into the subdomains and starting with the northern Alpine rim, the strongest change of the diurnal cycles (top row in Fig. 4) is found in June, for which the lightning activity between 18 and 06 UTC (19-07 LST) increased by about 50% after 2000 compared to the time before. The relative changes for the same periods in May are on the order of 25%. August reveals decadal variability with the 1990s and 2000s having been less active than the 1980s and the 2010s. These changes in the diurnal cycles affect the annual cycle for this region (left panel in Fig. 5). The lightning season begins earlier after 2000. The second half of the lightning season has decadal variability but no clear trend.

In the high Alps, the diurnal cycles of lightning changed strongly between May–Aug (middle row in Fig. 4). The increase is most prominent in June, for which the probabilities doubled. Also in May the increase is close to doubling. In July and August the increase is also pronounced, but the relative change is not as strong as in May and June. The resulting disparate annual cycles (center panel in Fig. 5) can be summarized as follows. In the 2010s the season starts earlier and lasts longer. The smoothed annual cycle crosses the 2.5% mark 30 days earlier in the 2010s than in the 1980s. Also, the seasonal peak is reached earlier and has an amplitude of more than one percentage point higher than in the 1980s and 1990s.

At the southern Alpine rim, lightning activity increases throughout the investigated season from May until September (bottom row in Fig. 4). Over the whole season relative changes of around 50% are not exceptional. As it was the case in the other subdomains, strong changes especially take place after the diurnal peak that extend lightning activity into the evening and night hours. The annual cycle reconstructed for the southern Alpine rim (right panel in Fig. 5) exhibits that the lightning season in the 2010s is stronger with an earlier onset and extended tail compared to the 1980s.

In the surrounding flatlands an increase over the past four decades of around 0.5–1.0 percentage points has been reconstructed for the second halves of the diurnal cycles from May until July (Fig. 4). The aggregation of the reconstructions to the annual cycles further reveals that lightning activity in the second half of the season has been lower in the 1990s.

### Climate trends

**Fig. 6:**
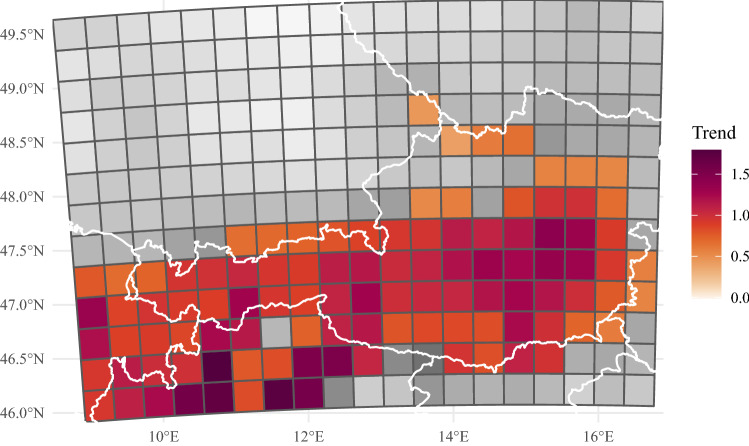
Linear climate trends: Color luminance gives the slope per decade of a linear regression for mean probability of lightning within an hour in percent. Desaturated colors in the grids indicate that the linear trends for these grids are not significant at the 5% level. Only June and afternoons between 13 and 19 UTC are considered

**Fig. 7:**
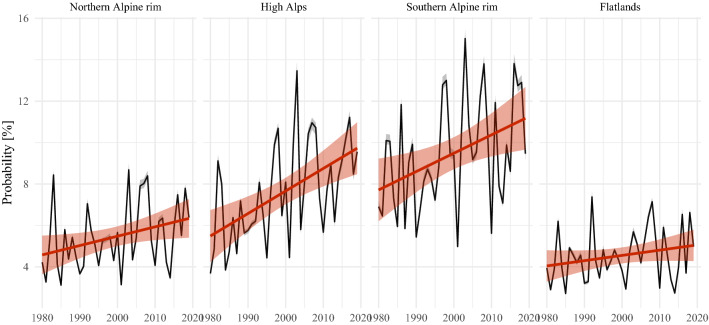
Linear trends for June afternoons (13–19 UTC) aggregated for the subdomains. The gray intervals around the black lines give the uncertainty obtained by the ten-fold cross-validation. The red intervals give the uncertainty of the liner fit

The analysis of diurnal and annual cycles reveals a strong increase of lightning activity especially in late spring and early summer. Therefore, further investigation of trends over the period 1980–2019 is performed. The focus will be on afternoon lightning between 13 and 19 UTC as this time of the day exhibits the largest absolute values for lightning probability in the whole domain. To investigate the trends, the reconstructed lightning probabilities for June afternoons are averaged for each year. Then the evolution of the aggregated probabilities over time is explained by linear regressions. The slope coefficients of the linear regressions serve as measures for the trends. Inference for the slope coefficients is provided by testing the hypothesis that the slope coefficient is not equal to zero against the null hypothesis that the slope coefficient is equal to zero. The results are first presented on the scale of the 32 km grid boxes (Fig. 6), and subsequently, on the scale of the four subdomains (Fig. 7).

Not a single cell in the whole domain has a negative trend. Almost all grids south of $47.5^\circ$ N show significant increase at the 5% level. Only at the very southeast where the topography flattens into plains the lightning trends are lower and insignificant. Another remarkable outcome is that more than a quarter of cells exceed an increase of one percentage point per decade.

The shape of the region with significant trends matches well the regions with complex topography. In particular the high Alps are witnessing large increases. The strongest trends reaching up to 2.0 percentage points per decade are found on the southern foothills in Italy. Another local hotspot is the region in the very east of the Alps between $14^\circ$ and $16^\circ$ E and around $47^\circ$ N. Trends North of the Alps in the Bavarian-Bohemian Forest around $14^\circ$ E and $48.5^\circ$ N are weaker but still significant and positive.

Aggregating the probabilities on the scale of the subdomains (Fig. 7) strengthens the results of the trend analysis. In the high Alps, the increase is highest with 1.1 percentage points per decade, which explains 29.4% of the variance in this time series. For the southern and northern Alpine rim, the analyses gives trends of 0.89 and 0.45 percentage points per decade, respectively. For these three domains the trends are significant at the 5% level. The trend for the flatlands of 0.25 percentage points per decade is not significant.

**Fig. 8:**
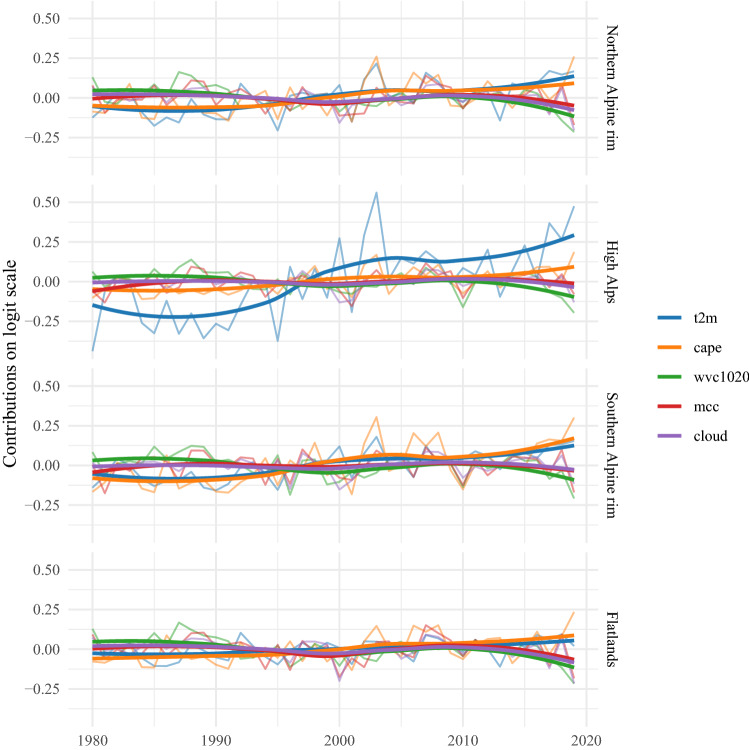
Mean contributions of additive terms on the logit scale for June afternoons (13–19 UTC) aggregated for the subdomains. The thin lines refer to values for individual years. For smoothing the thick lines show a local polynomial regression fit to the yearly values

The additive form of the statistical model (Eq. 1) allows one to further analyze the contributions of the individual functions $g_*()$ to $\text {logit}(\pi )$. Note that the additive contributions act on the logit scale, not on the probability scale. Figure 8 shows the mean contributions of the five most important additive terms for June afternoons. They are the contributions to the trends shown in Fig. 7. The strongest trend was found for the high Alps. The second panel in Fig. 8 reveals that the 2 m-temperature has a dominating contribution to this trend in the statistical model. For the Northern and Southern Alpine rim, 2 m-temperature and CAPE equally contribute to the reconstructed increase in lightning activity.

## Discussion

The lightning-MOS approach successfully replicates diurnal and seasonal cycles of observed cloud-to-ground lightning and reconstructs lightning for pre-measurement periods. Three recent advances contribute to its success: Long enough high-quality and homogeneous lightning measurements, physically consistent atmospheric conditions extending prior to lightning records, and statistical/machine learning techniques to weave the two together, correct the weaknesses of the atmospheric reanalysis and apply their good out-of-sample predictive skill to extend lightning records into the past.

Lightning strikes are locally rare events. Even at the peak of convective season and time of day, probabilities for the occurrence linger mostly in the single digits (cf. Figs. 2, 3, 4 and 5) and spatio-temporal variation is high. However, the additive nature of GAMs allows adding information from grid cells that share spatial characteristics (location, altitude), temporal characteristics (diurnal and seasonal), and the state of the atmosphere to achieve a successful replication of the lightning measurements. GAMs allow a *functional* dependence on all these variables. The shape of these functions is directly derived from the data and thus potentially non-linear. This flexibility permits to properly account for the diurnal cycle of lightning, which simple, single proxy variables (Finney et al. 2014; Taszarek et al. 2021, among others) cannot accomplish. This flexibility is also sufficient to achieve good out-of-sample performance with “only” a decade’s worth of lightning measurements as the cross-validation (Sect. 4.2) demonstrates. The additive and functional nature of GAMs makes it possible to incorporate approximations of the effects of multiple processes known to lead to lightning that can be derived from the available location, temporal and atmospheric state information—including favorable environments, triggers and electrical charge separation.

GAMs also compensate for weaknesses of the observational data. They allow to extend the relatively short duration of automated and spatially continuous lightning measurements, derive phenomenona that are not resolved in the reanalysis data, and correct the somewhat misrepresented diurnal cycle of convection in ERA5. As the blue curves for CAPE conditional on convective precipitation in Fig. 2 show, convection in ERA5 has a premature peak and too strong an amplitude, a result that Watters et al. (2021) also found. Yet with a GAM (red curves in Fig. 2), phase and amplitude become well reproduced.

All models presented here have to rely on the assumption that the functions mapping the explanatory variables (ERA5) to the target variable (ALDIS cloud-to-ground lightning occurrence) are stationary. This requires both the explanatory and the target variables to be homogeneously detected/modelled over time. The period for the lightning data (2010–2019) has been selected, to avoid effects of upgrades in the detection hardware and software. ERA5, spanning 40 years here, is by design physically consistent, but its accuracy is influenced by the number and kind of observations assimilated, which has changed over time and can potentially have noticeable consequences (Gleixner et al. 2020). This inherent limitation of ERA5 has to be kept in mind while looking at the trends found in this study.

The proposed technique using a flexible regression model to describe lightning is in general transferable to other regions (Ukkonen and Mäkelä 2019). For the new region lightning observations and model outputs have to be available in sufficient amount and quality as the regression model has to be re-fitted to the new domain. One advantage of this re-fitting is that the regression model learns processes specific to the new region.

Besides reconstructing lightning activity down to diurnal and annual cycles and analyzing trends, the additive structure of the GAM (Eq. 1) enabled the investigation of the contributions of the individual functions $g_*()$. The processes captured by lightning-MOS include favorable environments for thunderstorms ($\texttt{cape}$ and $\texttt{cp}$), charge separation ($\texttt{cswc2040}$ and $\texttt{wvc1020}$), and triggers ($\texttt{ishf}$). The investigation of the contributions revealed that in the high Alps the 2 m-temperature explains the majority of the increase, followed by CAPE. At the northern and southern rim these two parameters contribute equally to the positive trends. This finding suggests a connection to the observation that the Alpine region is stronger affected by climate warming than the remainder of Europe (Kuhn and Olefs 2020; Brugnara 2020).

Finally, one has to note that using cloud-to-ground flashes in the development of the lightning-MOS necessarily means that the resulting reconstructions have no way of knowing about or addressing any potential climate-related effect on the more abundant intracloud lightning. This is in particular important with respect to the production of NO$_x$ and ozone by lightning (DeCaria et al. 2005; Zhang et al. 2020).

## Conclusion

Lightning in the European Eastern Alps is probabilistically reconstructed back to 1980 by building a generalized additive model (GAM, Wood 2017) that takes atmospheric reanalysis output (ERA5, Hersbach et al. 2020) to explain the occurrence of lightning from the lightning location system ALDIS (Schulz et al. 2016). To honor the roots of this approach in the model output statistics (MOS) post-processing of numerical weather prediction models (Glahn and Lowry 1972), we call our approach *lightning-MOS*.

The hourly resolution of ERA5 allows one to reconstruct and investigate diurnal cycles of thunderstorms. Further, its high vertical resolution including that of cloud micro-physics makes it possible to incorporate a wide range of atmospheric processes. The lightning-MOS outperforms a baseline climatology and reconstructs the diurnal and annual cycles more accurately than state-of-the-art proxies for convective environments.

The reconstruction over 40 years (1980–2019) reveals amplification of the diurnal and annual cycles. This is especially pronounced in spring in the high Alps where lightning activity doubled, and at the southern Alpine rim throughout the season. A trend analysis reveals that the strongest trend is found in the high Alps. This sensible region is prone to climate change as measurements show that the warming signal is stronger in this region compared to other parts of Europe (Kuhn and Olefs 2020; Brugnara 2020).

## Data Availability

The ERA5 data are accessible via the Copernicus Climate Change Service (C3S) Climate Data Store. ALDIS data are available on request from OVE-ALDIS (aldis@ove.at)—fees may be charged.

## Footnotes

1 L137 model level definitions: https://www.ecmwf.int/en/forecasts/documentation-and-support/137-model-levels.

## Declarations

### Code availabliity

The results in this study were achieved using R, a software environment for statistical computing and graphics. The add-on packages **mgcv** and **bamlss** (Umlauf et al. 2021) were used for statistical modeling. We used the netCDF4 data format.
